# Splicing in the Diagnosis of Rare Disease: Advances and Challenges

**DOI:** 10.3389/fgene.2021.689892

**Published:** 2021-07-01

**Authors:** Jenny Lord, Diana Baralle

**Affiliations:** ^1^School of Human Development and Health, Faculty of Medicine, University of Southampton, Southampton, United Kingdom; ^2^Wessex Clinical Genetics Service, University Hospital Southampton NHS Foundation Trust, Southampton, United Kingdom

**Keywords:** splicing, RNA, diagnostics, rare disease, RNA-seq

## Abstract

Mutations which affect splicing are significant contributors to rare disease, but are frequently overlooked by diagnostic sequencing pipelines. Greater ascertainment of pathogenic splicing variants will increase diagnostic yields, ending the diagnostic odyssey for patients and families affected by rare disorders, and improving treatment and care strategies. Advances in sequencing technologies, predictive modeling, and understanding of the mechanisms of splicing in recent years pave the way for improved detection and interpretation of splice affecting variants, yet several limitations still prohibit their routine ascertainment in diagnostic testing. This review explores some of these advances in the context of clinical application and discusses challenges to be overcome before these variants are comprehensively and routinely recognized in diagnostics.

## Introduction

Diagnosis of rare disease has been revolutionized over the last decade. Advances in sequencing technologies have made large-scale exome and whole genome sequencing projects feasible. Analytical pipelines which rapidly call variants, annotate and prioritize those most likely to cause disease are well established. These advances have led to huge improvements in diagnostic rates and a rapid increase in the number of genes underlying rare diseases being identified. However, even with whole genome sequencing, often around 50% of patients do not receive a diagnosis (Mattick et al., 2018). There are likely many different reasons for these “missed” diagnoses, including the underlying cause of the condition not being detected (e.g., through poor sequencing coverage, or being of a type that is less easy to identify, such as complex structural rearrangements), or the cause being detected, but not recognized as being causal. The latter will include variants in genes not yet associated with a disorder and variants with functional consequences that are not captured by standard variant filtering approaches. Recent modeling estimated over 1,000 developmental disorder genes remain to be discovered, but may be increasingly difficult to identify due to issues of reduced penetrance and high pre- or perinatal mortality (Kaplanis et al., 2020). Filtering of candidate diagnostic variants tends to focus on protein coding consequences. Stop-gained and frameshift variants that lead to loss of function, canonical splice site, and predicted pathogenic missense variants are all generally prioritized, while other classes of variants, such as those affecting promotors or splicing (which may be deep intronic, near splice-site, synonymous, or missense) are often missed. Our incomplete understanding of elements governing gene regulation and splicing limits our ability to identify and interpret the effects of such variants.

**Figure 1:**
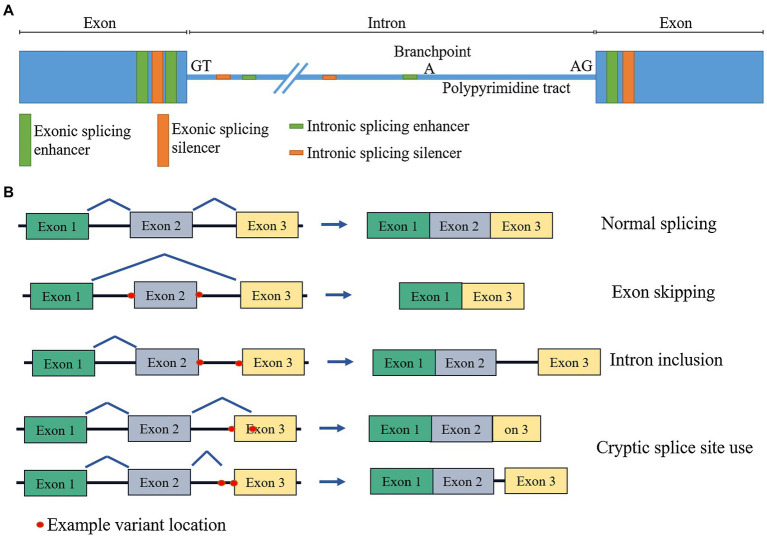
Schematic diagram of **(A)** cis splicing regulatory elements and **(B)** effects of splicing disruption on mRNA.

Variants which affect splicing are significant contributors to human disease. Splicing is the process by which introns are removed, and exons joined together in pre-mRNA processing. It is mediated by a large RNA–protein complex (the spliceosome), reliant on numerous cis and trans acting factors. Cis elements include the splice acceptor and donor sites themselves (the two nucleotides immediately flanking the exon, often termed the “essential” or “canonical” splice site), the polypyrimidine tract (a string of C and T nucleotides upstream of the acceptor site), the branchpoint (an A nucleotide, upstream of the polypyrimidine tract, which is the point of lariat formation during the splicing reaction), as well as exonic and intronic splicing enhancers and silencers (Figure 1). Trans factors include a host of protein and RNA accessory molecules that bind elements in the pre-mRNA and mediate spliceosome assembly and function. It is a complex and tightly regulated process, which can be disrupted by perturbations to any of these elements, damaging existing splice sites or regulatory elements, or generating new ones that outcompete the old. The mechanisms of splicing are yet to be fully understood, which negatively impacts our ability to ascertain whether a variant will disrupt splicing.

A number of advances in recent years have brought us closer to the aim of being able to accurately identify and classify potentially splice disrupting variants. This review presents an overview of several of these advances, explores implications for clinical practice, and identifies areas for further improvement.

## Identification of Potentially Splice Disrupting Variants

The mainstays of genetic diagnosis over the past decade have been targeted gene panel testing, and more recently, exome sequencing. Both of these methods have inherent limitations which restrict their ability to identify pathogenic variants. Panel testing involves the targeted sequencing of the coding regions of selected genes known to be associated with a given disorder. This relies on a comprehensive knowledge of the disease in question’s etiology, since genes missing from the panel will not be sequenced and therefore causal variants within these will be missed. Exome sequencing avoids this issue of incomplete prior knowledge, since it involves enrichment and sequencing of all protein coding genes. While a virtual panel-based analysis approach is often utilized in identifying potentially pathogenic variants, the sequence for all genes is generated, so newly identified disease-associated genes can be analyzed and diagnostic yields can be significantly increased through iterative reanalysis of data (Wright et al., 2018). Both of these approaches, however, focus on the coding regions of genes, with limited coverage of intronic regions beyond the canonical splice sites, prohibiting detection of splice disrupting variants in deeper intronic regions. There are also issues around highly variable coverage, with repetitive sequence, GC-rich regions, incomplete probe design, and short read sequence alignment limitations, all contributing to imperfect coverage of protein coding exons (Meienberg et al., 2015; Barbitoff et al., 2020), which leads to variants in and around poorly captured regions being missed.

Whole genome sequencing, which is increasing in popularity and usage as costs decrease, circumvents many of these issues. Coverage across the full genome is theoretically comprehensive across both coding and non-coding regions (although technical limitations still prevent coverage of certain areas; Ebbert et al., 2019) and more uniform than with exome sequencing (Barbitoff et al., 2020). However, the ever-expanding use of next-generation sequencing in diagnostics has brought a major challenge for researchers and clinicians in that the identification of variants outstrips interpretation of their effects, with each exome or genome sequenced generating numerous variants of unknown significance (VUS). A variant’s frequency in the general population, mode of inheritance, and predicted functional impact can be used to triage those most likely to be pathogenic, but there are many potentially diagnostic variants that are typically lost during standard diagnostic filtering pipelines that may lead to the true diagnosis being missed. This includes many potentially splice affecting variants that occur outside of the canonical splice site, in the near-splice region, as well as exonic and deeper intronic variants. Intronic variants in particular pose a problem, as due to lower selective constraint acting in these regions, many more intronic than exonic variants exist, and determining which of these may be functionally important *via* experimental validation is prohibitively expensive and time-consuming without effective triage of the most likely candidates.

## Classification of Potentially Splice Disrupting Variants

### *In silico* Predictions

Numerous different tools exist to predict the impact of variants on pre-mRNA splicing, but there is little consensus on optimal usage, limiting their implementation in the research and clinical settings. Under ACMG guidelines, predictions from *in silico* tools may be used as supporting evidence in variant classification if multiple lines of computational evidence suggest a variant has a deleterious effect (Richards et al., 2015). They can also be used to prioritize potentially splice disrupting variants for functional testing/experimental validation.

For successful integration into a clinical setting, *in silico* splicing prediction tools must meet several criteria. Firstly, the tool must reliably predict the functional impact of variants, which will be discussed below. Secondly, the tool must be easy to implement and the output easy to interpret. In a clinical setting in particular, there is benefit to having an intuitive graphical user interface or interactive web service, rather than accessing a tool *via* the command line, as bioinformatics expertise may be limited. Additionally, a single integrated platform for prediction of splicing abnormalities, as well as other types of variation (e.g., missense variants), is likely to be beneficial to streamline analysis. This likely contributes to the popularity of the Alamut software (Interactive Biosoftware, Rouen, France) in clinical laboratories, which enables the integrated analysis of multiple types of variation by drawing on multiple external sources of data, including gnomAD (Karczewski et al., 2020), ClinVar (Landrum et al., 2018), SIFT (Ng and Henikoff, 2003), and MutationTaster (Schwarz et al., 2010). Several tools for splicing prediction are integrated into a dedicated *in silico* splicing prediction window, including MaxEntScan (Yeo and Burge, 2004) and NNSPLICE (Reese et al., 1997) for predicting disruption to, or gain of, canonical splice sites, and ESEFinder (Cartegni et al., 2003) and RESCUE-ESE (Fairbrother et al., 2002) for the analysis of exonic splicing enhancers. Alamut enables easy comparison of wild-type and variant sequences and can generate a report on splice sites or splicing enhancers. Analysis with Alamut, however, has a relatively low throughput, and a recent comparison showed the tools available in Alamut (v2.11) were less accurate in prediction of the impact of functionally validated variants, either individually or in combination, than the newer, machine learning-based splicing prediction tool, SpliceAI (Jaganathan et al., 2019; Wai et al., 2020).

SpliceAI is a deep neural network for predicting the impact of variants on splicing trained on pre-mRNA (unspliced) sequence alone (Jaganathan et al., 2019). Jaganathan et al. initially trained the model with input sequence lengths of 80, 400, 2,000, and 10,000 nucleotides, with the 10,000 nucleotide (SpliceAI-10K) model providing the greatest predictive power, indicating that it was capable of detecting long-range determinants of splicing. The scores generated by the tool range from 0 to 1, with the value reflecting the probability of disruption to splicing. In the assessment of performance by Wai et al., the predictive ability of various tools was tested on 257 variants which had been experimentally validated for splicing impact. SpliceAI achieved an overall accuracy above 90% (Wai et al., 2020).

SpliceAI is not unique in attempting to harness the power of machine learning in predicting the impact of variants on splicing. Recent years have seen a huge increase of machine learning-based tools for splicing prediction, with an overview of methods and approaches recently reviewed by Rowlands et al. (2019). Many of these tools perform well in the small number of studies that have compared their usage. In addition to Wai et al., Rowlands et al. also found SpliceAI to be the single best predictive tool in their assessment of 9 tools’ performance on 250 VUS, although they did report greater accuracy from a weighted combination of multiple prediction tools, highlighting a strategy to improve predictions further, since combining multiple tools may mitigate individual weaknesses (Rowlands et al., 2021a). Surveying GT>GC splice donor variants, Chen et al. tested SpliceAI’s ability to distinguish those which disrupt splicing with those that maintain normal splicing since these variants are not universally disruptive (Chen et al., 2020). SpliceAI was able to distinguish variants which did and did not generate wild-type transcripts with accuracies ranging from 33 to 89% across the three datasets utilized, while a previous study had found numerous other widely used tools were largely unable to do so (Lin et al., 2019). SpliceAI was also found to outperform other tested methods by Riepe et al., who tested the predictive power of 13 different tools on deep intronic and near-splice variants in two genes associated with Stargardt disease (*ABCA4*) and cardiomyopathy (*MYBPC3*; Riepe et al., 2021). SpliceAI gave the most accurate predictions for both deep intronic (94% accuracy) and near-splice variants (79% accuracy) in *ABCA4*, although SpliceSiteFinder-like (Shapiro and Senapathy, 1987) was found to perform better on near splice variants in *MYBPC3* (74% accuracy, vs. SpliceAI’s 67%).

There has been a high degree of variance in SpliceAI’s performance across different variant sets (ranging from 67 to 94% in Reipe et al. and 33–89% in Chen et al.). This variance may be due to relatively limited sample sizes or may reflect genuine differences in predictive power across different genes, variant types, and contexts. Many of these comparisons are somewhat limited in their scale and scope and have generally compared SpliceAI with older tools rather than other machine learning based approaches. It will take time and testing on a wide range of genes and variants to establish true accuracy and potential for diagnostic use. Machine learning-based approaches can clearly achieve high predictive accuracy, but ascertaining the precise degree of trust and confidence that can be placed in predictions is crucial for diagnostic application and establishing this is a relatively undefined process. Independent, large-scale testing across a variety of different clinically relevant scenarios is needed to estimate true clinical utility. The Critical Assessment of Genome Interpretation (CAGI) project may provide the right platform for this. CAGI was set up with the aim of improving genomic variant interpretation by establishing state of the art prediction strategies for different types of variants. Participants are invited to submit predictions for variants with validated but unpublished effects, giving a relatively unbiased test of predictive performance. Having previously included relatively little in the way of splicing variant interpretation, the 5th edition of CAGI included two large-scale splicing prediction challenges—Vex-seq and MaPSY (for a comprehensive overview, see Mount et al., 2019).

Variant exon sequencing, or Vex-seq (Adamson et al., 2018), is a high-throughput approach which was applied to test the splicing impact of 2,059 single nucleotide variants (SNVs) and short indels from the Exome Aggregation Database (ExAC; Lek et al., 2016) using a minigene-based reporter system. Results were quantified as the change in exon inclusion vs. the wild type, termed delta Percent Spliced In (ΔPSI). The CAGI challenge was to make a quantitative prediction of the ΔPSI for the variants tested. The Massively Parallel Splicing Assay, or MaPSY (Soemedi et al., 2017), which comprised the second CAGI5 splicing challenge, assessed the splicing impact of reported pathogenic exonic mutations, again using a minigene based system. Here participants were invited to predict whether or not each variant caused skipping of constitutively included exons. Five groups participated in each challenge, with predictions based on a variety of different machine learning approaches and utilizing varied datasets to assist in training of models (Mount et al., 2019). Overall, a novel method, MMSplice, or modular modeling of splicing outperformed other and existing tools in its predictions (Cheng et al., 2019a,b; Mount et al., 2019). MMSplice is based on a modular set of neural networks trained separately for acceptor and donor sites, exons and intronic regions, and utilizing diverse data sources, including gene annotations from GENCODE (Harrow et al., 2012) and functional information on variants from massively parallel reporter assays (Cheng et al., 2019b). Combining the separate modules allows assessment of the splicing impact of variants from 50 bp upstream of acceptor sites, throughout exons, and to 13 bp downstream of donor sites with high accuracy across all of these regions (Cheng et al., 2019b; Mount et al., 2019).

MMSplice has recently been further expanded with the addition of a neural network for modeling tissue-specific regulation, with this new tool termed Multi-tissue Splicing (MTSplice; Cheng et al., 2021). Although most variants affecting splicing were found to have consistent effects across tissues, the group did report gains in accuracy from combining the tissue specific module, particularly in brain tissue. Models with increased complexity, such as this, may help further refine *in silico* predictions. Large datasets of paired genome and multi-tissue RNA sequencing, massively parallel splicing reporter assays, and CLIP-seq maps of splicing regulatory elements provide exciting opportunities to further expand splicing prediction models.

As important as the choice of tools for splicing prediction is the approach to interpreting the output from them. Usually, a score or probability is given reflecting the likelihood that a given variant affects splicing. The choice of what threshold to use as a cutoff to classify a variant as (predicted) splice affecting is often relatively arbitrary and/or based on assessment against a relatively small number of variants with known outcome. In research, choice of threshold may be tailored to meet the needs of the study, favoring sensitivity or specificity depending on study design, but in the clinic, a unified and evidence-based approach is badly needed.

The 6th edition of CAGI is set to occur in summer 2021, providing an excellent opportunity for consistent and unified assessment of splicing tools, which may help to drive these powerful new methods closer to widespread clinical use.

### Experimental Confirmation

Since sufficiently reliable *in silico* tools have yet to be properly established in the clinical diagnostic setting, experimental validation to confirm the effect of variants on splicing is important in gaining a definitive diagnosis. Over many years, RT-PCR and minigene-based experimental confirmations have been used to test splicing disruption. Minigenes are circular plasmids containing everything needed for expression. They feature exons flanking an intron, into which can be inserted a region of interest. For splicing assessment, the region of interest (typically an exon and short flanking intronic sequence) is inserted into the plasmid, both in reference and variant form, such that after expression in a cell line, the splicing of the wild-type and alternative forms can be compared to assess the effect of the variant on splicing. Minigenes have the benefit that they can be used in almost any genomic context, and do not rely on the availability of patient samples, since variants can be generated artificially, e.g., with site directed mutagenesis. However, the artificiality of the construct removes much of the larger context in which the variant occurs, so results may not reflect the true picture of splicing within the patient. RT-PCR for splicing assessment avoids this issue, since the subject of study is mRNA from the patient. Here, patient-derived mRNA is converted to cDNA before being amplified with primers designed to capture the impact of variants on splicing. This is then compared against controls with electrophoresis and/or sequencing. Availability of patient samples from disorder appropriate tissues can be a limiting factor in its utility, although blood-based RT-PCR has successfully been used to assay splicing in genes with extremely low expression in blood, and for which blood would not be an obvious disease-relevant tissue (Wai et al., 2020).

Both methods have been used extensively in the characterization of splicing variants over the years, testing splicing disruption from a variety of variant types (intronic, missense, synonymous) across many different genomic contexts (Gaildrat et al., 2010; Sharma et al., 2014; de Calais et al., 2017; Dionnet et al., 2020; Wai et al., 2020). While successful in research laboratories, their widespread use in clinical diagnostics is hindered by their relatively low throughput nature, since minigenes and RT-PCR primers must generally be tailored to each variant to be tested. This also means a prior candidate variant, or at least gene is needed for this kind of testing, limiting their use in discovering new diagnoses without strong prior expectation of the cause.

### RNA-seq Assessment of Splicing

Transcriptome sequencing, or RNA-seq, offers many benefits over longer-standing RT-PCR and minigene validation methods. This method allows the total RNA content of a given sample to be surveyed in a single experiment, instead of single variant or gene testing. Several studies have shown RNA-seq methods have significant promise for increasing diagnostic yields—in clarifying the effects on splicing of VUS and detecting splicing perturbations even when there is no known candidate variant.

One of the first major studies using transcriptome sequencing for rare disease diagnostics came from Cummings et al. (2017). The group explored the potential diagnostic uplift from the application of RNA-seq in rare muscle disorders. Muscle disorders are particularly amenable to this type of investigation, as muscle tissue is relatively easily biopsied so issues surrounding differential splicing and expression across different tissues are avoided. The group sequenced muscle tissue mRNA from 63 patients with suspected monogenic muscle disorders and analyzed the data alongside 184 control samples from The Genotype-Tissue Expression (GTEx) project (The GTEx Consortium, 2013). Thirteen patients were already diagnosed with variants suspected to affect the transcriptome and acted as positive controls to test the methodology, while the remaining 50 either had a candidate splicing variant (*n* = 4), a strong candidate gene (*n* = 12), or no candidate gene or variant (*n* = 34). The group devised their own analytical pipeline, Mendelian RNA-seq, to identify, normalize, and filter for likely pathogenic splicing events, as well as testing for evidence of allelic imbalance (different levels of expression from the two alleles of a gene due to, e.g., regulatory variants). An overall diagnostic rate of 35% was achieved in the cohort, with the highest yields for those with a suspected candidate variant (50%) and those with a strong candidate gene (66%), but even with no prior candidate gene or variant, causation could be established in 21% of cases.

In 25 patients with muscular dystrophy lacking a molecular diagnosis after exome/panel sequencing, Gonorazky et al. adopted a similar strategy, using a modified version of the Mendelian RNA-seq approach introduced by Cummings et al. to identify splicing abnormalities, as well as looking for evidence of differential expression and allelic imbalance (Gonorazky et al., 2019). A diagnosis was established in 36% of cases (9/25), with the majority of those affecting splicing.

For mitochondrial disorders Kremer et al. used RNA-seq in RNA from fibroblast cell lines derived from muscle biopsies to attempt to diagnose 48 unsolved patients (Kremer et al., 2017). LeafCutter (Li et al., 2018) was used to identify aberrant splicing events, with each sample being compared to all others, and samples were also tested for evidence of aberrant and mono-allelic expression. An overall diagnostic rate of 10% was established (5/48 patients), with three of these disrupting splicing, while candidate genes were identified for a further 75% of patients (36/48). LeafCutter, the tool used in this study, is a sensitive and highly scalable method which allows annotation-free quantification of RNA splicing from short-read RNA-seq data (Li et al., 2018). More recently, an expansion of the algorithm has been released, termed LeafCutter for Mendelian Disease (LeafCutterMD) specifically for the purpose of detecting splicing outliers (Jenkinson et al., 2020). Compared to the earlier implementation and “one versus all” approach used by Kremer et al., LeafCutterMD was found be more resilient to noise in the data and to have greater power to detect outlier splicing events. Reanalysis of previously generated data with new methodologies may therefore help to identify further diagnoses.

These studies demonstrate notable gains in diagnostic yields can be obtained through RNA-seq. Common features of the studies include the use of disease-relevant tissues and a focus on disorders with relatively concise causal gene lists. Having a small number of potentially causal genes reduces the search space for splicing investigations when there is not a known candidate variant. In Cummings’ study, a median of 105 candidate splice disruption events per proband was detected across all genes—a prohibitively high number for manual review in a large-scale study. When restricting to all OMIM disease-associated genes, this number was 26, further reduced to a median of just five candidate events per proband for known neuromuscular disease-associated genes. Likewise, a median of 5 candidate splicing events per proband was detected in Gonorazky’s neuromuscular gene panel. This is a tenable number of variants for manual assessment of evidence supporting the abnormal splicing event and consideration of the flagged gene as a diagnostic candidate for the patient in most contexts. For disorders with much larger numbers of potentially causative genes, or patients where an appropriate candidate gene panel is unclear, other strategies may need to be adopted to prioritize likely pathogenic events to avoid analyses being dominated by irrelevant splicing changes.

Splicing is known to be highly tissue specific (Yeo et al., 2004), and as such selecting a disease-relevant tissue to survey for splicing may be crucial in detecting causal aberrant events, as genes may not be expressed in other tissues, or patterns of splicing and splicing disruption may differ. Muscle disorders are therefore good candidates for RNA-seq for splicing abnormalities, as muscle is one of the few routinely clinically biopsied tissue types. Kremer et al. used fibroblasts derived from muscle biopsies in their investigation of mitochondrial disorders caused by disruption of nuclear genes. Most genes involved in mitochondrial function are well expressed in other tissues (Kremer et al., 2017), including in fibroblasts, so despite physiological effects of mitochondrial gene disruption in fibroblasts likely being minimal, they can act as a proxy tissue for more clinically relevant tissues such as the brain, heart, and skeletal muscle whose high energy demands make mitochondrial dysfunction particularly pronounced.

The requirement for a clinically relevant tissues does, however, limit the spectrum of disorders for which RNA-seq can be conducted. Skin and muscle biopsies are relatively easily obtained, but are still somewhat invasive. Blood is arguably the most commonly taken clinical sample and may be disease relevant for some disorders. Urine may be used as a source of epithelial cells from the kidney and urinary tract (Molinari et al., 2018). For many rare diseases, such as the multitude of neurodevelopmental disorders whose key manifestations lie within the brain, there is no easily obtainable tissue that is clinically relevant. For splicing analysis *via* RNA-seq to be viable in such cases, alternative RNA sources must be utilized.

Frésard et al. sequenced RNA from whole blood in a cohort of 94 patients with undiagnosed rare diseases, across a spectrum of disorders, including neurology, musculoskeletal and orthopedic, hematology, and ophthalmology, for many of which blood would not be an obvious tissue of interest (Frésard et al., 2019). The group noted that 76% of a panel of 284 genes implicated in neurological disorders, and 66% of all loss of function intolerant genes showed some expression in blood. Overall, a diagnostic yield of 7.5% was observed across the cohort, with candidate genes flagged for a further 16.7% based on a combination of expression, splicing, and allele-specific expression analyses. Although this is a lower diagnostic yield than seen in the previously discussed studies, it shows a broader applicability of the technology as several candidates were identified for patients with neurological conditions, for which blood would not be an obvious choice of target tissue.

In 115 undiagnosed patients with a diverse range of phenotypes, Murdock et al. undertook RNA-seq of both whole-blood and skin fibroblasts (Murdock et al., 2021). They were able to achieve a diagnostic rate of 17% across patients without diagnoses from exome or genome sequencing (14/82), and 12% across the full cohort, some of whom were diagnosed *via* DNA analysis. In comparing the utility of RNA-seq in the two different tissues, they found fibroblasts gave higher and more consistent expression of disease-relevant genes, with only immunodeficiency related genes showing greater expression in blood (Murdock et al., 2021).

To facilitate the choice of appropriate tissue for RNA-seq analyses, online resources have been developed to compare expression and splicing of genes of interest across different tissue types. Panel Analysis of Gene Expression (PAGE) was introduced by Gonorazky et al. and is available as an online platform^1^ (Gonorazky et al., 2019). PAGE allows comparison of gene and exon expression and splicing patterns across different tissues, utilizing the GTEx data. Users can search for a gene, or gene panel of interest and compare across tissues to establish the most appropriate tissue to capture the RNA profile of interest. In a similar vein, MAJIQ-CAT (available at https://tools.biociphers.org/majiq-cat/) was developed to help users establish appropriate clinically accessible proxy tissues for RNA-seq analyses (Aicher et al., 2020). The study classed whole blood, Epstein–Barr virus transformed lymphocytes, and fibroblasts as clinically accessible tissues (CATs) and compared these to a further 53 non-CAT tissues using expression and splicing data. The group found 40.2% of genes in non-CAT tissues were inadequately represented by at least one of the CATs, although just 6.3% of genes were inadequately represented by all CATs. For the majority of genes that were inadequately represented by at least one CAT, low levels of expression were responsible, although for 5.8% of genes per non-CAT, the gene was adequately expressed and captured by the sequencing analysis, but the pattern of splicing was sufficiently different that the CATs were not reliable proxies. This valuable resource can guide the choice of proxy tissue for clinicians and researchers and also help clarify the limitations of RNA-seq by understanding which genes will not be adequately represented by the chosen proxy.

As well as the choice of tissue impacting the likely success rate of diagnostic RNA-seq, there are many other considerations to take into account in terms of study design and analytical approach. A full overview of such considerations is beyond the scope of this review, but some aspects particularly relevant to splicing analyses are briefly discussed below. Chhangawala et al. explored the effect of sequencing read length on both differential expression analysis and splice junction detection, and while for differential expression they found little impact of increasing read length from 50 to 100 bp, for splice event detection, the impact was marked (Chhangawala et al., 2015). Use of 100 bp read length was associated with a significantly greater number of splice junctions being detected relative to shorter read lengths, and paired end reads outperformed single end. Indeed, Mapleson et al. explored the same question with reads up to 200 bp and found splice junction discovery improved in both recall and precision as read lengths increased (Mapleson et al., 2018). Some of this improvement is due to more reliable alignment of longer, paired end reads to the reference genome, although some benefit also likely comes from longer reads being more likely to traverse splice junctions and have overhanging fragments of sufficient length to be reliably placed, a feature many junction detection methods exploit when calling splicing events. Related to this, sequencing depth will also determine how comprehensively splicing can be profiled. A study targeting genes well expressed in the chosen tissue will need fewer sequencing reads to survey splicing than a study with genes that are poorly expressed. A recent preprint by Rowlands et al. presents a web tool^2^ that can be used to calculate the minimum required sequencing depth to profile splicing based on the level of expression of genes of interest in three different CATs, which will assist in choosing the appropriate sequencing depth (and target tissue type) in study design (Rowlands et al., 2021b). An additional consideration here is that disruption to splicing often leads disruption of the transcript’s open reading frame and the introduction of a premature termination codon. When this occurs, transcripts are generally targeted for nonsense mediated decay, a process by which cells eliminate problematic transcripts to prevent the production of truncated proteins. In such instances, the transcripts of interest will be undergoing active degradation, so will be present with lower abundance than would otherwise be expected, and will require greater sequencing depth to be adequately surveyed.

In terms of analysis strategies, many different tools exist for detecting aberrant splicing events from RNA-seq, and as yet, there is not a clear consensus about which tool, or combination of tools is the most appropriate, and this may vary depending on the precise aims of the experiment. Broadly speaking, these tools depend on the detection of split reads—those that span exon/exon boundaries—to infer where splicing has occurred, quantify splice site usage, and compare this between individuals or groups. Mehmood et al. recently compared the performance of a number of different methods for differential splicing detection (Mehmood et al., 2019), grouping the tools in to different categories based on the approach they take (exon-based, isoform-based, and event-based). Overall, the best performance across a range of metrics was seen for exon-based methods [e.g., DEXSeq (Anders et al., 2012), edgeR (Robinson et al., 2010)] and two event-based methods [e.g., MAJIQ (Vaquero-Garcia et al., 2016), rMATS (Shen et al., 2014)]. A striking finding of the study, however, was that there was very little concordance between results given by different tools, leading the authors to recommend the use of multiple tools when analyzing RNA-seq to maximize discovery of events and detection of aberrant splicing.

Overall, using RNA-seq to investigate differences in expression and splicing shows real promise for increasing diagnostic yields in rare disease cohorts where exome and genome sequencing have failed to detect or clarify the underlying causation. Diagnostic yields in early studies range from 7.5–36%, even when the tissue sampled is not obviously linked to the disorder in question. Diagnostic yields have so far been higher where the disease-affected tissue is the one that is sequenced, and where a relatively small number of genes of interest are surveyed, reducing false positives and the requirement for extensive manual assessment of ultimately irrelevant splicing events. Platforms such as MAJIQ-CAT which enable users to assess appropriate alternatives where the affected tissue is not clinically accessible will help broaden the applicability of the technology. Improvements in methodologies for detecting and prioritizing aberrant splicing events in RNA-seq data are needed to decrease the number of candidate variants for the technology to be effectively applied across the entire transcriptome in a gene agnostic manor.

Care must be taken in the interpretation of splicing variants, even where functional assessment is conducted. Numerous examples of conflicting variant interpretations and changes in interpretation over time exist in the literature, and splicing variants appear to be enriched for these reclassifications relative to many other variant types (Esterling et al., 2020). This has been particularly well documented in familial cancer syndromes associated with *BRCA1* and *BRCA2* variants, where pathogenic variants are clinically actionable. The *BRCA1* variant, c.594-2A > C, observed in cis with c.641A > G, was initially observed to cause skipping of exon 10, giving a frameshift effect, and thus identifying the variant as pathogenic (Tesoriero et al., 2005). Later analyses, however, demonstrated although the majority of transcripts contained this truncating disruption, it was likely due to the linked variant, c.641A > G, and around 20–30% of transcripts had a loss of both exons 9 and 10. This generates a naturally occurring alternative splicing product that maintains the reading frame and likely produces a BRCA1 protein which retains tumor suppressor function, rendering these linked variants unlikely to be pathogenic (de la Hoya et al., 2016). A recent study of four *BRCA2* variants by Nix et al. discussed the complexities of splicing variant interpretation. Under ACMG guidelines (Richards et al., 2015), each could be classified as pathogenic, or likely pathogenic, and three had ClinVar (Landrum et al., 2018) entries with these classifications. Careful consideration of functional evidence, however, revealed partial splicing abnormalities, likely functional in-frame transcripts, and GT>GC splice site maintaining alterations, suggesting these variants are actually likely benign (Nix et al., 2021). These studies demonstrate the importance of thorough functional characterization, the value of gene specific knowledge, and show the fluidity of variant interpretation over time.

## How Prevalent is Disease Caused by Splicing Mutations?

Diagnostic yields can clearly be increased by incorporating splice-affecting variants that may currently be overlooked, such as near-splice, deep intronic, and synonymous variants, but the extent of this diagnostic uplift is difficult to quantify at present. As variants which do not disrupt the essential splice sites are not captured routinely by standard variant prioritization strategies, a systematic picture of their contribution is lacking. There are two related questions that will help to address this which have been the subject of significant research in recent years—what proportion of variants disrupt splicing, and what proportion of variants that do disrupt splicing lie outside of the commonly ascertained 2 bp essential splice sites, so are currently under appreciated. Numerous attempts to quantify these values have been made over the years. With estimates for the former ranging from ~15% to over 60% (Krawczak et al., 1992; López-Bigas et al., 2005), there is clearly a large degree of uncertainty here.

It is often cited that up to 50% of all disease-causing variants disrupt splicing, based on estimates from the *ATM* and *NF1* genes (Teraoka et al., 1999; Ars et al., 2000). In 80 patients with neurofibromatosis type 1, Ars et al. screened cDNA for variants in *NF1*, detecting 44 unique mutations in 52 patients. Of these, 50% were shown to disrupt splicing, including essential and near splice, missense, and nonsense variants. Teraoka et al. examined 62 variants in *ATM*, causal of ataxia-telangiectasia, and found ~50% of these affected splicing. In both of these studies, the majority of splice disrupting variants did not fall within the essential dinucleotides, suggesting over half of splicing mutations may be missed by the current focus on essential splice site variants. Both of these estimates, however, are based on relatively low numbers of variants in single genes from cohorts of patients with specific disorders, and as such are somewhat biased in their ascertainment and do not give a reliable picture of the overall proportion of variants affecting splicing across the human genome.

As mentioned previously in the context of CAGI, Soemedi et al. provided data on a much larger number of disease-ascertained variants in their 2017 study (Soemedi et al., 2017). The group selected 4,964 exonic disease-associated mutations from HGMD (Stenson et al., 2003) and developed MaPSy to test effects of the variants on normal splicing, using reporter constructs with mutant or wild-type exonic and intronic sequence, both *in vitro* (incubation with nuclear extract) and *in vivo* (transfection into human embryonic kidney cells). Where patient tissue samples were available to validate the method, there was a high degree of concordance (26/32, 81%) with the MaPSy findings. Overall, 10% of the surveyed exonic variants altered splicing in both the *in vivo* and *in vitro* test systems.

Massively parallel functional testing for splicing effects is a promising approach for quantifying the proportion of variants that disrupt splicing. Several examples have been published in recent years using saturation mutagenesis to assay all possible single nucleotide changes, generally focusing on short exons in disease-associated genes. Mueller et al. surveyed the impact of all possible synonymous variants in *SMN1* exon 7 using a minigene containing exons 6–8, with shortened introns in between (Mueller et al., 2015). The group found 23% (32/138) of synonymous variants reduced the inclusion of exon 7 to a level of 70% of the wild-type allele or lower [70% was selected as the cutoff here as patients with spinal muscular atrophy (SMA) type III exhibit ∼70% exon 7 inclusion vs. the wild type]. These findings are largely in agreement with those of Souček et al., who also used a modified minigene system to survey the impact of all single nucleotide changes in the same exon (Soucek et al., 2019). Here, of 181 assayed variants, 58 showed a significant increase in skipping of *SMN1* exon 7 (32%), but only 37 of these (20%) reduced inclusion by >1%, so the majority of variants had very small effect sizes.

In the gene *RON*, Braun et al. randomly mutated minigenes to assess alternative splicing of exon 11 (Braun et al., 2018). Transcripts missing exon 11 are associated with increased tumor invasiveness (Collesi et al., 1996). The minigenes, which spanned exons 10–12, including full introns, incurred an average of 3.6 variants, with 97% of positions mutated at least ten times, and a linear regression approach was adopted to infer the impact of individual variants. 45% of constructs showed a change in inclusion level >10%, with the regression predicting over 90% of all positions in exon 11, and at least 50% of flanking intronic and exonic positions had at least one variant that altered isoform usage >5%.

Ke et al. employed a similar approach, assessing the splice affecting potential of all single and double nucleotide changes in *WT1* exon 5 (Ke et al., 2018). Overall, 70% of all variants affected splicing at least twofold, with around 33% leading to increased and 37% leading to decreased exon inclusion. For SNVs, around 65% of variants affected splicing, while for double mutations, this figure was around 72%.

For *FAS* exon 6, an alternatively spliced exon, Julien et al. were able to assay all single nucleotide changes for effects on splicing (Julien et al., 2016). The minigene they constructed including *FAS* exons 5–7 was transfected into human embryonic kidney cells which were cultured in conditions to promote approximately 50% exon inclusion levels from the wild type, allowing decreased as well as increased inclusion to be surveyed. Of 189 possible SNVs, 39% showed a decrease in exon inclusion, 22% showed an increase in exon inclusion, and 39% did not affect exon inclusion levels, with similar proportions for synonymous and non-synonymous changes. Overall, variants at 92% of positions assayed impacted splicing. The high proportion of splice affecting variants observed here is somewhat at odds with the previous reports.

Cheung et al. developed an orthogonal system to assess the impact of genetic variation on exon recognition at scale, termed multiplex functional assay of splicing using Sort-seq (MFASS; Cheung et al., 2019). MFASS is a reporter system based on constructs of 3 exons and two introns, where skipping of the central test exon reconstitutes fluorescence. The construct undergoes site-specific integration into human cells, ensuring a single construct per cell, and fluorescence-activated cell sorting is used to divide the cells into bins based on fluorescence levels, and thus exon inclusion levels. DNA sequencing is used to identify constructs, with normalized read counts being used to generate an inclusion index for that construct. After optimization and validation of the method on known splicing regulatory elements, the group used MFASS to assess the splicing impact of 27,733 naturally occurring, mostly rare variants seen in ExAC. They found that 3.8% of variants caused almost total loss of exon recognition, and while these disruptive variants were enriched at the acceptor and donor sites, the majority of disruptive variants fell outside these regions.

Table 1 provides a summary of the findings of these studies for single variants. The high variance in estimates of the proportion of variants that disrupt splicing (∼4–65%), even in studies with relatively similar designs, highlights the challenges associated with interpreting splicing variants and quantifying the likely diagnostic uplift from increased ascertainment of these variants. There are several limitations common to many of these studies that may limit their generalizability to the landscape of splicing regulation genome wide. Firstly, many of the studies focus on short exons as these are more easily assayed by these methods (e.g., *SNM1*’s exon 7 is just 54 bp, while the average human exon is around 120 bp). Splicing regulatory elements have been shown to be enriched toward the ends of exons (Savisaar and Hurst, 2017), so the focus on short exons which enriches the target region for positions close to the splice site may lead to over estimation of the proportion of positions that when altered affect splicing. Exons are also often selected on the basis of being disease relevant, which may bring biases of its own, although enhances the utility of the studies as they may assist in interpreting VUS clinically. Second, many of the studies do not utilize the full native context of the exon in question (i.e., exon surrounded by full flanking introns and exons). The splicing regulatory landscape is known to be complex and diffuse, so removing the full local context is likely to remove more distal regulatory information, possibly leading to an artificially inflated reliance on regulatory sequences within the exon itself. In fact, some studies (e.g., Braun) artificially alter the normal exonic or flanking sequence in order to promote instability and create a volatile genetic environment where variants are more likely to impact splicing, perhaps overinflating estimates of the proportion of variants that disrupt splicing and limiting generalizability to the human genome as a whole.

**Table 1:** Proportion of single nucleotide variants (SNVs) that disrupt splicing across studies.

Study	Target region	Variants of interest	Variants assayed (*n*)	Variants affecting splicing beyond given threshold (%)
Teraoka et al.	*ATM*	Disease associated	62	50
Ars et al.	*NF1*	Disease associated	44	50
Soemedi et al.	Various	HGMD disease associated	4,964	10
Mueller et al.	*SMN1* exon 7	Synonymous variants	138	23
Souček et al.	*SMN1* exon 7	All SNVs	181	20
Julien et al.	*FAS* exon 6	All SNVs	189	60
Braun et al.	*RON* exon 11	All SNVs (linear regression)	1,800	43
Ke et al.	*WT1* exon 11	All SNVs	141	65
Cheung et al.	Various	ExAC variants, mostly rare	27,733	3.8

Another key difference in the study’s methodologies is in the threshold used for determining whether a variant affects splicing. Mueller’s study used 70% of the wild-type inclusion levels as their threshold for determining splice effects, based on SMA type III patients exhibiting this level of inclusion, but for other studies, the rationale for selecting a threshold was less clear, and indeed, the level of splice perturbation that is necessary for function to be disrupted is an unanswered question and is likely to vary gene by gene, or even exon by exon. It is also possible that the differences in the results of these studies reflect the genuine biological picture, and that the proportion of splice disrupting variants does indeed vary widely across different genomic contexts. In a recent paper, Baeza-Centurion raised the possibility that one of the main determining factors in the proportion of variants that have splice disrupting potential may be the normal level of expression of the exon in question, with constitutively expressed exons showing greater resilience to splice disruption, while the majority of splice disrupting variants impact exons which are already alternatively spliced (Baeza-Centurion et al., 2020). Since the majority of expressed exons in human genes are highly expressed [around 60% of exons expressed in >10% of transcripts are actually included in >90% of transcripts (Baeza-Centurion et al., 2020)], the generalizability of these experiments largely focused on lower expressed exons may again be limited. In instances where a variant causes splicing disruption to an already alternatively spliced exon, clinical interpretation is particularly challenging. Sensitivity to altered inclusion levels of alternatively spliced exons is likely to vary between exons, genes, and contexts (e.g., tissue type), making determination of pathogenicity extremely complex.

A final way to approach understanding of the likely diagnostic uplift from increased appreciation of splicing variants is to assess the prevalence of non-essential splice site mutations relative to the more easily ascertained essential splice site mutations. Zhang et al. and Lord et al. looked at mutational intolerance surrounding splice sites, identifying particular near splice positions particularly sensitive to splice disruption when mutated (Lord et al., 2018; Zhang et al., 2018). Using data from GTEx and Guevadis, Zhang et al. were able to demonstrate that carriers of variants in constrained positions had fewer correctly spliced reads than those with reference homozygous genotypes (Zhang et al., 2018). They estimated that by taking variants in these constrained positions into account, around 35% more splicing variants would be found relative to just essential splice variants (Zhang et al., 2018). Based on relative proportions of *de novo* variants in essential and near splice positions, Lord et al. estimated 27% of splice disrupting variants to fall in near splice positions rather than essential splice positions (Lord et al., 2018). This value had been estimated at ~30% previously by Krawczak et al. based on data from HGMD (Krawczak et al., 2007) and 43% using data from Caminsky et al. (2014). All of these estimates are reasonably consistent, although are likely an underestimate of the true contribution of non-essential splice site variants as ascertainment of splice disrupting variants at significant distance from splice sites has been inconsistent historically. When considering variants in ClinVar (Landrum et al., 2016), Lord et al. found that just 16.5% of pathogenic and likely pathogenic variants fell in near splice positions, with 83.5% in the essential splice dinucleotides, highlighting the under-ascertainment of these variants in clinical databases (a deficit estimated to be ~35–40%, highly consistent with the estimate from Zhang et al.; Lord et al., 2018). Jung et al. recently reported their comprehensive characterization of abnormal splicing events caused by intronic variants in human cancers and found 46% of splice disrupting events were caused by deep intronic variants, with 35% of these activating cryptic splice sites (Jung et al., 2021). These findings are likely to be echoed in the rare disease setting, demonstrating huge diagnostic potential in greater ascertainment of this class of variants.

Taken together, these studies, along with the diagnostic uplift from RNA-seq, suggest increased ascertainment of non-essential splice disrupting variants stand to increase diagnostic yields by at least 35%. The much higher estimates from some of the massively parallel splicing assay approaches may reflect the fact that although splicing is altered by a higher proportion of variants than this, the disruption is at a level, or having a functional impact, that is not clinically relevant. The level and nature of disruption necessary for clinical presentation may vary by gene or by exon. Haploinsufficient disease genes are likely to be less resilient to splicing disruption in general than recessive disease genes, where the expression of some normally spliced transcripts may be enough to prevent disease. Variants affecting splicing have been linked to low/partial penetrance in several disorders, due to the production of both normal and aberrantly spliced transcripts (Rave-Harel et al., 1997; Schubert et al., 1997; Arrabal et al., 2011). In patients with cystic fibrosis, the intron 8 5T variant in *CFTR* has been shown to generate both normal transcripts and transcripts in which exon 9 is skipped. Rave-Harel et al. demonstrated that the relative expression of these two transcripts was correlated with disease severity, with a variety of lung pathologies linked to lower expression of the full-length transcript. Interestingly, they also noted the proportions of correctly spliced transcripts varied between the nasal and epididymal epithelia, with the epididymal epithelia showing lower levels of the properly spliced form in infertile males (Rave-Harel et al., 1997). The same disruptive variant showed high variability in levels of correct splicing between individuals, and between tissues within individuals, highlighting the complexities of interpreting splicing variants in rare disease. The functional impact of any altered splicing may also have a profound impact on clinical variant interpretation. Splicing aberrations where the reading frame is maintained may be less severe than frameshifting changes where nonsense mediated decay is likely to occur. This has been observed to be the case with variants in the *DMD* gene, with splicing variants that maintain reading frame (e.g., triggering skipping of a single in frame exon) leading to the milder Becker Muscular Dystrophy, while variants that disrupt the reading frame lead to the more severe Duchenne Muscular Dystrophy, which is being exploited for therapeutic purposes (Mendell et al., 2013, 2016). In-frame splicing changes do still have the potential to be disease causing, particularly if a skipped region is crucial for protein structure or function (e.g., encodes a binding or catalytic domain). All of these factors further complicate efforts to estimate an overall figure for splicing disruption in rare disease.

## Challenges Remaining

Despite the advances discussed, there are still many challenges that remain for comprehensive detection of splice disrupting variation to be standard in clinical diagnosis. Proper validation of *in silico* tools for diagnostic use will facilitate triage of the variants most likely to be functionally disruptive and may in the future be reliable enough to avoid the need for expensive experimental characterization. RNA-seq holds great promise for detecting splice disruption in patient samples, but further work is needed to establish the best practices for this. Choice of target tissue and sequencing design will have major impacts on ability to detect splicing disruption. Optimization and standardization of RNA-seq analysis methodologies for splicing detection are badly needed. Guidance on the level of splicing disruption that is needed for pathogenicity is required, and this may be highly context/gene dependent. Careful curation of splicing variants in clinical databases will be needed to minimize errors in interpretation and ensure the widest possible benefit is gained from functional and clinical assessment. We are also still limited by our knowledge of the mechanisms of splicing. The interdependent signals that govern correct splicing are yet to be fully understood, limiting prediction and interpretation of variants. Machine learning, which has shown great promise in predicting the effects of variants, may be of further use in this area if we can disentangle the features these predictions depend on to gain new knowledge of the splicing code.

## Conclusion

The advances and improvements discussed in this review show we are closer than ever to being able to comprehensively identify and establish the effects of variants affecting splicing. The ascertainment of these variants in the clinic stands to have a major impact on diagnostic yields across rare disease. Many challenges still remain, but with the increase in splice-modifying therapeutics (Finkel et al., 2017) offering hope for patients and families affected by rare disorders, there has never been greater motivation to overcome these issues and see splicing diagnostics and therapeutics truly integrated in to clinical care.

## Author Contributions

JL and DB conceived the idea and content. JL drafted the manuscript with editing and final approval by both JL and DB. All authors contributed to the article and approved the submitted version.

### Conflict of Interest

The authors declare that the research was conducted in the absence of any commercial or financial relationships that could be construed as a potential conflict of interest.

## Footnotes

^1^https://page.ccm.sickkids.ca/

^2^https://mcgm-mrsd.github.io/
